# Protocol for Prevention and Monitoring of Surgical Site Infections in Implant-Based Breast Reconstruction: Preliminary Results

**DOI:** 10.3390/medicina57020151

**Published:** 2021-02-08

**Authors:** Giovanni Papa, Andrea Frasca, Nadia Renzi, Chiara Stocco, Giuseppe Pizzolato, Vittorio Ramella, Zoran Marij Arnež

**Affiliations:** Plastic and Reconstructive Surgery Unit, Department of Medical, Surgical and Health Sciences, University of Trieste, 34100 Trieste, Italy; giovanni.papa@asugi.sanita.fvg.it (G.P.); nadia.renzi@asugi.sanita.fvg.it (N.R.); chiara.stocco@asugi.sanita.fvg.it (C.S.); giuseppe.pizzolato@asugi.sanita.fvg.it (G.P.); vittorio.ramella@asugi.sanita.fvg.it (V.R.); zoranmarij.arnez@asugi.sanita.fvg.it (Z.M.A.)

**Keywords:** breast reconstruction, implant, infection, prevention, antibiotic prophylaxis, complication

## Abstract

Surgical site infection in implant-based breast reconstruction is a complication with variable incidence reported in the literature. Due to potential loss of implant and reconstruction, it can have a strong psychological impact on patients. *Background and objectives:* This study aimed primarily at analyzing the current status of the surgical site infection (SSI), (type, time of onset, clinical presentation, pathogens and management) in patients who underwent implant-based breast reconstruction at our Breast Unit. Secondarily, we wanted to establish whether introduction of a new, updated evidence-based protocol for infection prevention can reduce SSI in implant-based breast reconstruction. *Materials and Methods:* A single-center retrospective study was performed primarily to evaluate the incidence and features of SSI after implant-based breast reconstruction from 2007 to 2020. In June 2020, a protocol for prevention of SSI in implant-based breast reconstruction was introduced in clinical practice. Secondarily, a data analysis of all patients who underwent implant-based breast reconstruction in compliance with this protocol was performed after preliminarily assessing its efficacy. *Results:* 756 women were evaluated after mastectomy and implant-based breast reconstruction for breast cancer. A total of 26 surgical site infections were detected. The annual incidence of SSI decreased over time (range 0–11.76%). Data relating to infections’ features, involved pathogens and implemented treatments were obtained. Since the introduction of the protocol, 22 patients have been evaluated, for a total of 29 implants. No early infections occurred. *Conclusions:* Surgical site infection rates at our Breast Unit are comparable to those reported in the literature. The SSI rates have shown a decreasing trend over the years. No SSI has occurred since the introduction of the prevention protocol for surgical site infection in June 2020.

## 1. Introduction

Surgical site infection (SSI) is one of the most common healthcare-associated infections (HAIs) and a major cause of increased hospital stay and mortality. SSI is a significant surgical complication in prosthetic breast reconstruction. The incidence reported in literature ranges from less than 1% up to 43% [1,2,3,4]. This variability can be explained by the absence of a unique definition, which could allow a diagnosis based on standardized criteria. According to the National Nosocomial Infection Surveillance System (NNIS), SSI is related to the surgical procedure and typically occurs within 30 days after surgery. In the case of implant-based breast reconstruction, this interval is prolonged to one year after surgery [5]. Three types of SSI are proposed by the Centers for Disease Control and Prevention (CDC): superficial incisional, deep incisional and organ and space SSI.

The clinical diagnosis of SSI is made by observing the classic signs of inflammation, (redness, delayed healing, fever, pain, tenderness, warmth, or swelling).

The risks for SSIs after the placement of prosthetic implants are multiple and have been extensively investigated [6,7,8,9,10,11]. They are related to the patient (age, smoking, obesity, diabetes mellitus, immunosuppression, presence of simultaneous infections or bacterial colonization, etc.) or to the surgical procedure (pre-operative shower and skin preparation, duration and repetition of hand washing, skin antisepsis, operative time, antibiotic prophylaxis, sterilization of surgical instruments, use of prosthetic material, drainage, intra-operative hypothermia, etc.)

SSI risk assessment is based on the National Healthcare Safety Network (NHSN) risk index [12], consisting of three equally weighted factors: the American Society of Anesthesiologists (ASA) score (3, 4, or 5), wound classification (contaminated or dirty), and operative time in minutes (>75th percentile). Each risk factor represents 1 point; thus, the NHSN SSI risk index ranges from 0 (lowest risk) to 3 (greatest risk).

The most frequently isolated pathogens in surgical site infections are *S. aureus* and Coagulase-negative staphylococci. However, the number of infectious complications due to multi-drug resistant (MDR) microorganisms is increasing, and the isolation of these pathogens in biological materials is associated with poor clinical outcomes. Among the multi-drug resistant (MDR) microorganisms, the most common ones include Methicillin-resistant *S. aureus* (MRSA), *Streptococcus*, and Gram-negative bacteria, such as *Pseudomonas* [13]. It has been shown that a large proportion of SSIs originate from the patients’ own flora. Nasal carriage of *S. aureus* is now considered a well-defined risk factor for subsequent infection in various groups of patients [14,15]. Several interventional studies have attempted to reduce the infection rates by eradicating nasal carriage (screening for *S. aureus*, nasal decolonization by mupirocin, skin decontamination) [16,17].

In implant-based breast reconstruction, SSI could lead to prolonged hospitalization, re-intervention, multiple outpatient checks, or even to the loss of the reconstruction. Infections in prosthetic reconstruction correlate with the increased incidence of capsular contracture [18], one of the main indications for surgical revision.

No consensus exists regarding the duration of antibiotic prophylaxis and whether it should be continued after surgery in the presence of a drain close to the implant or in selected high-risk patients. A customized approach to this issue seems to be the most appropriate; in fact, some patients with certain risk factors such as diabetes mellitus, obesity, or low-quality mastectomy flaps can benefit from prolonged antibiotic administration [19].

The primary aim of our study is to analyze the current status of infection rates at our institution (type of infection, timing of onset, clinical manifestations, pathogens involved, and potential treatments) in patients undergoing prosthetic breast reconstruction. The secondary aim is to evaluate the effectiveness of a new Prevention Protocol for SSI by analyzing the patients’ data.

## 2. Materials and Methods

### 2.1. Retrospective Analysis

We conducted a monocentric study at the Plastic and Reconstructive Surgery Unit of Azienda Sanitaria Universitaria Giuliano Isontina (ASUGI)—Trieste to retrospectively review SSIs’ rates and characteristics among patients undergoing prosthetic breast reconstruction (immediate direct-to-implant and two-stage tissue expander/implant breast reconstruction) between January 2007 and June 2020.

The analyzed SSIs’ characteristics include the type of diagnosed infection, the interval between surgery, the diagnosis of infection, the symptoms associated with infection, the pathogens isolated from microbial cultures, and the number of revision surgeries.

### 2.2. Prospective Analysis

We prospectively enroll all patients undergoing implant-based breast reconstruction from the introduction of our Prevention Protocol for SSIs (June 2020) in a prospective study aimed at evaluation of its effectiveness.

Prevention Protocol for SSIs involves a pre-operative phase, reported in Box 1, concerning MSSA/MRSA screening (Methicillin-sensitive *S. aureus*); an intra-operative phase with several advices to observe during surgery, resumed in Box 2; and antibiotic therapy timing outlined in Box 3.

This protocol has been applied in a standardized way to all patients operated for breast cancer and reconstructed using prosthetic implants, from June 2020 on. We considered the type of mastectomy; the overall duration of the surgical procedure; eventual use of acellular dermal matrices; post-operative complications; the need for revision surgeries.

Box 1. **Prevention Protocol for SSIs—Pre-operative phase.**

**PRE-OPERATIVE PHASE**

Screening for MSSA/MRSA

(up to 6 weeks prior to surgery):

- Nostrils swab
- Cutaneous (axillary and perineal) swab

Decolonization:

- Body washing with chlorhexidine 4% (daily, from 3 days before surgery)
- Intraoral washing with chlorhexidine oral rinse (on the day of surgery)

Eradication if tested positive for MSSA:

- Body washing with chlorhexidine 4% (daily, from 3 days before surgery)
- Mupirocin 2% nasal ointment (applied three times daily, from 3 days before surgery)

Eradication if tested positive for MRSA:

- Body washing with chlorhexidine 4% (daily, from 5 days before surgery)
- Mupirocin 2% nasal ointment (applied three times daily, from 5 days before surgery)
- Re-screening 48–72 h after eradication protocol *

MSSA, Methicillin-sensitive *S. aureus*; MRSA, Methicillin-resistant *S. aureus*; SSIs, surgical site infections. * It is mandatory to have 3 negative screenings before surgery, done at a time frame of 7 days or more after the eradication protocol, which could be administered maximum twice; if the patient keeps being tested positive for MRSA, administer adequate intravenous antibiotic prophylaxis before surgery and if possible, isolate the patient.

Box 2. **Prevention Protocol for SSIs—Intra-operative phase.**

**INTRA-OPERATIVE PHASE**

- Surgical hand preparation with antimicrobial soap and water or alcohol-based hand rub before donning sterile gloves
- Preparation of the skin prior to draping using 2% chlorhexidine with 70% isopropyl alcohol
- Perform careful atraumatic pocket dissection and careful haemostasis
- Change surgical gloves every 60’ to 90’ and before handling implants
- Perform pocket irrigation *
- Minimize implant open time to reduce contamination
- Use a “minimal or no-touch” technique where possible
- Use closed suction drains to reduce hematoma or seroma formation in selected cases, “tunneling” them into a subcutaneous plane
- Warming devices should be used to prevent hypothermia
- It is recommended to reduce the operating time
- Laminar airflow ventilation system

* There is a paucity of data supporting one form of washout to another. At our institution, we perform pocket and implant washing with antiseptic antibacterial 50% betadine double-antibiotic solution.

Box 3. **Prevention Protocol for SSIs—Antibiotic prophylaxis.**

**ANTIBIOTIC PROPHYLAXIS**

Intravenous antibiotic prophylaxis at the time of induction, for every patient:

- Cefazolin 2 g;
- OR Clindamycin 600 mg, if penicillin or cephalosporins allergies;
- Vancomycin 15 mg/kg + Gentamicin 3 mg/kg, if patient positive for MRSA

Intravenous 24-h multiple-dose antibiotic prophylaxis:

- Cefazolin 1 g q8hr;
- OR Clindamycin 600 mg q8hr, if penicillin or cephalosporins allergies;

Prolonged post-operative antibiotic prophylaxis, in high-risk patients:

- Cefalexin 500 P.O. q6hr;
- OR Clindamycin 300 mg P.O. q8hr, if penicillin or cephalosporins allergies

P.O., oral administration.

The collected data were inserted and analyzed in two different databases: the retrospective included patients who developed SSI between January 2007 and June 2020 and the prospective which included patients from June to September 2020.

The data were collected and managed using Microsoft Excel (Microsoft Office 365). Descriptive statistic was performed using IBM SPSS Version 24.

## 3. Results

### 3.1. Retrospective Amalysis

In the period between January 2007 and June 2020, a total of 756 patients underwent surgical procedures involving prosthetic material for breast reconstruction. Twenty-six patients were diagnosed with SSI during the first year of follow-up after surgery.

Two out of 26 were Superficial incisional SSIs (7.7%); 24 out of 26 were Deep incisional SSIs (92.3%). No Organ or Space SSIs were reported (see Table 1).

Fifteen out of 26 SSIs had an early (within 30 days from surgery) onset (57.7%), 11 out of 26 SSIs had a late (between 31 days and 1 year after surgery) onset (42.3%); the median onset of SSI was 19 days after surgery.

Clinical signs, related to the onset of SSI, have not uniformly manifested in all 26 patients: we recorded local signs, such as redness, tenderness, warmth, or swelling in 24 out of 26 patients; fever was reported in 10 cases; purulent fluid discharge was reported in 7 cases. Clinical signs were supported by the evidence of inflammation markers increase C-reactive Protein (CPR) or Erythrocyte sedimentation rate (ESR) in 25 cases.

The organisms isolated from microbial cultures included: *S. aureus* was in 7 cases; *S. epidermidis* in 8 cases; other Coagulase-negative staphylococci in 2 cases; Gram-negative bacteria in 5 cases, Actinobacteria in two cases, and Fungi in one case. One patient had cultures done, but with negative bacterial growth.

At first, an empiric IV antibiotic treatment with βlactam ± inhibitor or Clindamycin was given in all SSIs pending cultures of wound’s swab or periprosthetic fluid collection. Later, targeted antibiotic therapy was administered: in 12 cases (46.2%), this was sufficient to contain and resolve the infection; in the remaining 14 cases (53.8%), a revision surgery was performed.

### 3.2. Preliminary Analysis after the Introduction of the Prevention Protocol for SSIs

From June 2020 to September 2020, we prospectively enrolled 22 patients. Nine cases were bilateral. Altogether, we treated 31 breasts. In two of the bilateral cases, a contralateral breast surgery for symmetry was performed without using implant (one breast reduction and one mastopexy). We placed a total of 29 prosthetic devices (tissue expanders or implants). Twelve cases were immediate breast reconstructions after mastectomy, involving either tissue expander or implant; 10 cases were tissue expander (or implant) replacements with potential contralateral breast surgery to achieve symmetry. Mean mastectomy flap thickness in patients undergoing immediate breast reconstruction was 16.2 mm (range, 4.7 to 36.4). Three out of 12 patients had poor implant flap coverage (flap thickness < 10 mm); 6 out of 12 had medium-thickness flap coverage (flap thickness between 10 and 20 mm); 3 out of 12 patients had good implant flap coverage (flap thickness > 20 mm). Mean implant (*n* = 20) size was 390 cc (range, 140 to 690 cc); mean tissue expander (*n* = 9) volume was 428 cc (range, 250 to 650 cc), and mean intra-operative inflated volume was 40% (range, 18 to 72%). More surgical details are reported in Table 2.

Mean age at surgery was 54.9 years (range, 28 to 75 years). Most patients were nonsmokers (90.9%). A total of 5 patients (22.7%) had risk factors such as diabetes mellitus, obesity, or immunosuppression status; only 1 patient had previous breast radiation therapy or postmastectomy radiation therapy. BRCA1 or BRCA2 positivity was noted in 3 patients (13.6%) (Table 3). 

Three out of 22 patients (13.6%) tested positive for MSSA (1 patient was nasal carrier, 1 patient was cutaneous carrier, 1 was both nasal and cutaneous carrier) and underwent eradication treatment. No patients in the study tested positive for MRSA. 

The mean follow-up period was 85.4 days (range, 33 to 129). At the moment, none of the enrolled patients had early post-operative SSIs.

Table 4 reports infection rates (%) from 2007 to 2020: it shows how the (early) infection rates dropped to 0% after the introduction of the Prevention Protocol for SSIs in June 2020. The results of late SSI for the year 2020 at this moment are unavailable.

## 4. Discussion

Surgical site infection (SSI) is one of the most common healthcare-associated infections (HAIs). SSI is a significant surgical complication in prosthetic breast reconstruction as it may lead to a longer hospital stay with increasing costs for the national health system. For the patient, it is a devastating complication when it ends with the loss of reconstruction.

There is a lack of evidence-based benefits of SSI prevention strategies in implant-based breast reconstruction. Breast implant infection rates reported in the literature range from less than 1% up to 43% [1,2,3,4]. This variability can be explained in part by the lack of a standardized definition. Moreover, infection rates are not always well documented. All these make performing of sufficiently powered studies to provide meaningful results difficult.

Over the years, different techniques have been introduced in order to improve aes-thetic and functional results in breast reconstruction. The use of prosthetic breast recon-struction has risen significantly, becoming the most frequent choice [20,21].

Patients undergoing implant-based breast reconstruction are subject to a range of infection prevention measures which are not standardized across institutions or countries. Actions to reduce SSIs have varying degrees of evidence for their efficacy, ranging from expert opinion to randomized trials, and are extremely debated.

Not unexpectedly, many hot topics and controversies in this field have emerged, including antibiotic prophylaxis, management of implant and pocket, early treatment of SSI.

Our study aimed to compare the infection rates related to implant-based breast reconstruction carried out at the Plastic and Reconstructive Surgery Unit of ASUGI—Trieste to those reported in the literature.

We reported a decreasing trend in SSIs’ rate over time (range, 0% to 11.76%). Since the introduction of the Prevention Protocol for SSIs in June 2020, no case of early infection occurred among patients undergoing implant-based breast reconstruction was noticed. Late infections require a one-year follow-up, so the results of the prospective study cannot be compared to the retrospective ones.

However, we remain confident that the decreasing trend of infection rate could continue and stand as close as possible to zero.

Our study reports 2 Superficial incisional SSIs (7.7%), 24 Deep incisional SSIs (92.3%), and no Organ or Space SSI. This trend reflects what has been reported in most of the studies and points out how SSIs related to prosthetic breast reconstruction rarely involve any area of the body other than skin, muscle, and surrounding tissue involved in the surgery [12].

Most early SSIs and implant failures are associated with endogenous skin flora that colonize the nipple, including *S. aureus*, streptococci, and lactobacilli species [13]. Our findings support this data, as we frequently isolated staphylococci from microbial cultures; *S. aureus* was identified in 7 cases; *S. epidermidis* in 8 cases; and other Coagulase-negative staphylococci in 2 cases.

Although most SSIs are generally thought to occur within a month, some occur later, even after many years [22,23]. We reported 42.3% of SSIs with late onset, occurring between 31 days to 1 year after surgery. We agree with Sinha et al. who, in 2017, showed, in a prospective multi-center trial, that 47–71% of total SSI complications occur as late infections and criticized data collection limited to a 30 day period following surgery, which significantly underestimates the risk of actual SSI in implant-based reconstructions [24].

The clinical spectrum of breast implant infection is highly variable. In our series, clinical signs have not uniformly manifested in all 26 cases: we recorded local signs, such as redness, tenderness, warmth, or swelling in 24 out of 26 patients; fever was reported in 10 cases; and purulent fluid discharge in 7 cases. Clinical signs were supported by a raise of the inflammation markers (CRP or ESR) in 25 cases.

The management of breast implant infection often involves tissue expander or implant removal and targeted intravenous antibiotic therapy for up to two weeks for common infections. Positioning of a new implant following removal can be attempted within 3–6 months, although this may not be possible in cases involving chest wall radiotherapy. In order to attempt salvage of prosthetic reconstruction, systemic antibiotics without implant removal may be successful in a subset of patients with mild SSIs [25,26].

SSIs in our series were managed in the first instance with empiric βlactam ± inhibitor or Clindamycin IV antibiotics treatment pending cultures of wound’s swab or periprosthetic fluid collection. Later, targeted antibiotic therapy was administered. Our data showed a 46.2% prosthesis salvage rate for SSIs that were treated with parenteral antibiotics only. Our salvage rate is comparable to those reported by other authors who have employed both surgical and medical treatments [27,28,29].

New antimicrobials, lipoglycopeptides, like dalbavancin, are long-acting antibiotics with potential for less frequent administration [30].

Patients undergoing implant-based breast reconstruction are exposed to a range of pre-operative, intra-operative, and post-operative prevention measures which are not standardized across institutions or countries, and which have varying degrees of evidence for their efficacy, ranging from expert opinion to randomized trials.

We created a standardized protocol for prevention of SSIs, based on international guidelines and evidence reported in the literature. We believe that it could be the starting point for further studies in the field of breast reconstruction. Only by creating a common pathway with standardized pre, intra and postoperative steps, we can study large populations, allowing more robust statistical analysis of complications and outcomes in breast reconstruction surgery.

Regarding the pre-operative management, MSSA and MRSA screening with appropriate treatment of carriers before surgery is recommended by several studies. General population carriage rates for *S. Aureus* are as high as 37.2%, and a carrier has a 7.1 relative risk of subsequently developing a related infection [14]. Our preliminary data show 13.6% MSSA carriage rate. Each patient underwent eradication treatment before surgery. No patients in the study tested positive for MRSA. Clearly, considering the commitment that requires a screening path, both in terms of personnel and materials, periodic revaluations will be necessary for a cost-benefit analysis, also based on the local prevalence of *S. Aureus*.

The retrospective nature of the analysis that was performed on infection rates at our institution has not allowed us to thoroughly analyze the risk factors involved. However, the data collection form that we have introduced along with the prevention protocol for SSIs will allow us to prospectively study the potential risk factors for each complication related to implant-based reconstruction.

Among these complications, SSI can cause devastating reconstructive failures in implant-based breast reconstructions; for this reason, the need for antibiotic prophylaxis remains one of the most debated topics. There is no consensus regarding the right duration of antibiotic prophylaxis after implant-based reconstruction, and whether it should be continued after surgery in the presence of a drain into the implant pocket or in selected high-risk patients (e.g., patients who had diabetes or recent radiation therapy).

Developing our protocol, we reviewed international guidelines [31,32], systematic reviews, and studies with high levels of evidence [17,33,34,35,36,37,38,39,40,41]. Prior to June 2020, all patients were subjected to prolonged antibiotic administration until drains removal. From June 2020, to all patients undergoing implant-based breast reconstruction we administered antibiotic prophylaxis extended to 24 h or longer in those patients deemed “high risk” for SSI, and as already pointed out, no case of infection occurred among them.

In clinical practice, there is a lack of standardization in terms of pre-, intra-, and post-operative care for patients undergoing implant-based breast reconstruction. Our new protocol shows excellent preliminary results in term of infection prevention.

Despite the limited sample size and relatively short prospective follow-up period not allowing for a statistically significant analysis of its effectiveness, the preliminary data, showing absence of early SSIs, could potentially lead to a decreasing trend also of late infection rates.

## 5. Conclusions

SSI is clearly a significant surgical complication in implant-based breast reconstruction as it may lead to a longer hospital stay with increasing costs for the national health system, and it may result in the loss of reconstruction, a potentially devastating complication for the patient.

Infection rates at our institution are comparable to those reported in the literature and show a decreasing trend over time. Additionally, since the introduction of the Prevention Protocol for SSIs in June 2020, no cases of infection were reported among patients undergoing implant-based breast reconstruction. As mentioned above, despite the limited sample size and relatively short prospective follow-up period not allowing for a statistically significant analysis of the effectiveness of this protocol, the preliminary data, with absence of early SSIs, could show a promising decreasing trend also of late SSI infection rates. We further believe that creation of a common shared pathway, with standardized pre-, intra-, and post-operative steps, represents the cornerstone for a valid and efficient treatment for the patient; moreover, it is also the starting point to carry out more robust analysis of complications and outcomes in implant-based breast reconstruction.

## Figures and Tables

**Table 1:** SSIs’ characteristics.

Variable	No. (%)
SSI classification	
Superficial incisional SSI	2 (7.7)
Deep incisional SSI	24 (92.3)
Organ or space SSI	0 (0)
SSI onset	
Early	15 (57.7)
Late	11 (42.3)
Pathogens	
*S. aureus*	7 (26.9)
*S. epidermidis*	8 (30.8)
Coagulase-negative staphylococci	2 (7.7)
Gram-negative bacteria	5 (19.3)
Actinobacteria	2 (7.7)
Fungi	1(3.8)
No bacterial growth	1 (3.8)
Outcome	
Need for revision surgery	14 (53.8)
Only antibiotic therapy	12 (46.2)

SSI, Surgical site infection.

**Table 2:** Surgical details.

Patient	Timing	Side	Left Side	Axilla	Reconstruction	Anatomical Plane	ADM	Right Side	Axilla	Reconstruction	Anatomical Plane	ADM	Operative Time (min)
1	IBR	Bilat	NSM	LNB	TE	Sub-pec	-	NSM	-	TE	Sub-pec	-	295
2	IBR	Bilat	NSM	-	Implant	Pre-pec	Braxon^®^	NSM	-	Implant	Pre-pec	Braxon^®^	226
3	IBR	Left	SSM	LFD	TE	Sub-pec	-	-	-	-	-	-	187
4	RPS	Bilat	-	-	TE to Impl	Sub-pec	-	-	-	TE to Impl	Sub-pec	-	189
5	IBR	Right	-	-	-	-	-	NSM	LNB	TE	Sub-pec	-	215
6	IBR	Left	SSM	LND	TE	Sub-pec	-	-	-	-	-	-	188
7	IBR	Left	SSM	LNB	TE	Sub-pec	-	-	-	-	-	-	174
8	IBR	Left	NSM	LNB	TE	Sub-pec	-	-	-	-	-	-	183
9	RPS	Left	-	-	TE to Impl	Pre-pec	-	-	-	-	-	-	70
10	RPS	Bilat	-	-	TE to Impl	Sub-pec	-	-	-	Breast Aug	Pre-pec	-	83
11	RPS	Right	-	-	-	-	-	-	-	TE to Impl	Sub-pec	-	130
12	RPS	Left	-	-	TE to Impl	Sub-pec	-	-	-	-	-	-	60
13	RPS	Left	-	-	TE to Impl	Pre-pec	-	-	-	-	-	-	70
14	IBR	Bilat	NSM	-	Implant	Pre-pec	Braxon^®^	NSM	LNB	Implant	Pre-pec	Braxon^®^	248
15	IBR	Right	-	-	-	-	-	SSM	LND	TE	Sub-pec	-	300
16	RPS	Bilat	-	-	Impl to Impl	Sub-pec	-	-	-	Mastopexy	-	-	150
17	IBR	Right	-	-	-	-	-	SSM	LND	Implant	Pre-pec	Braxon^®^	180
18	RPS	Bilat	-	-	Aug-Pexy	Pre-pec	-	-	-	TE to Impl	Sub-pec	-	170
19	IBR	Bilat	NSM	-	Implant	Pre-pec	Braxon^®^	NSM	-	Implant	Pre-pec	Braxon^®^	270
20	IBR	Right	-	-	-	-	-	SSM	LNB	TE	Sub-pec	-	190
21	RPS	Left	-	-	TE to Implant	Sub-pec	-	-	-	-	-	-	65
22	RPS	Bilat	-	-	TE to Implant	Sub-pec	-	-	-	Breast Red	-	-	162

IBR, Immediate Breast Reconstruction; RPS, Replacement surgery; NSM, Nipple-sparing mastectomy; SSM, Skin-sparing mastectomy; LNB, Lymph node biopsy; LND, Lymph node dissection; TE, Tissue expander; TE to Impl, Tissue expander replacement with Implant; Impl to Impl, Implant replacement with Implant; Breast Aug, Breast Augmentation; Aug-Pexy, Mastopexy with breast augmentation; Breast Red, Breast Reduction; Pre-pec, pre-pectoral plane; Sub-pec, sub-pectoral plane; ADM, Acellular dermal matrix.

**Table 3:** Patients’ demographics.

Variable	No. (%)
Mean Age	54.9
Smoking	
Current smoker	2 (9.1)
Nonsmoker	20 (90.9)
Comorbidities	
Obesity (BMI ≥ 30 kg/m^2^)	1 (4.5)
DM	3 (13.6)
IS	2 (9.1)
BRCA+	3 (13.6)
Previous XRT	1 (4.5)
MSSA carrier	3 (13.6)
MRSA carrier	0 (0)

BMI, Body Mass Index; DM, Diabetes Mellitus; BRCA, Breast-Related Cancer Antigens; XRT, Radiation therapy; IS, Immunosuppression status; MSSA, Methicillin-sensitive *S. aureus*; MRSA, Methicillin-resistant *S. aureus*.

**Table 4:** Infection rates.

Year	SSI (n)	Patients (n)	SSI %
2007	0	21	0
2008	0	17	0
2009	2	18	11.11
2010	2	17	11.76
2011	1	33	3.03
2012	4	56	7.14
2013	3	56	5.36
2014	2	48	4.17
2015	3	67	4.48
2016	1	76	1.32
2017	3	94	3.19
2018	2	105	1.90
2019	2	101	1.98
January–May 2020	1	47	2.13
**June–September 2020**	**0**	**22**	**0**

Table 4. Surgical site infection (SSI) rates. Please note that the infection rate for the year 2020 considers only early surgical site infections. Highlighted in bold is the period from June to September 2020 related to the introduction of the Prevention Protocol for SSIs.
